# Detection of micro-metastases by flow cytometry in lymph nodes from patients with penile cancer

**DOI:** 10.1186/s12894-018-0399-3

**Published:** 2018-10-05

**Authors:** Lu Zhang, Jin Hu, A. Ali Zirakzadeh, Jesper Rosvall, Mats Hedlund, Ping Sheng Hu, Robert P.A. Wallin, Amir Sherif, Ola Winqvist

**Affiliations:** 1 Department of Medicine, Immunology and Allergy Unit, Karolinska University Hospital, SE-171 76 Stockholm, Sweden; 2 Department of Urology, South General Hospital, Karolinska Institutet, Stockholm, Stockholm Sweden; 3 Cancer Biotherapy Center, Affiliated Hospital of Guizhou Medical University, Guiyang, China; 4 Department of Surgical and Perioperative Sciences, Urology and Andrology, Umeå University, Umeå, Sweden

**Keywords:** Penile cancer, Tumor draining lymph nodes, Flow cytometry, Micro-metastasis detection, Pan-cytokeratin AE1/AE3

## Abstract

**Background:**

The tumor draining lymph node concept was first described in penile cancer for staging. Immunohistochemistry and histopathology evaluations are routinely used in clinical practice to examine lymph nodes for metastasis. However, these methods are time-consuming with low diagnostic accuracy and micro-metastases might be missed. In this study, we aim to evaluate detection of metastatic cells in draining lymph nodes by flow cytometry.

**Methods:**

To assess the sensitivity of micro-metastasis detection by FACS (Fluorescence-activated cell sorting), HeLa cells were titrated into Peripheral blood mononuclear cells (PBMCs) and expression of pan-cytokeratin AE1/AE3 was analyzed. Single cell suspensions were separately prepared from 10 regional lymph nodes obtained from 5 patients with invasive penile cancer undergoing radical surgery and lymph node dissection. Lymph node dereived cells were examined for cell surface expression of EpCAM, E-cadherin and intracellular expression of pan-cytokeratin AE1/AE3 by FACS.

**Results:**

Ten lymph nodes from 5 penile cancer patients were investigated in a head-to-head comparison between FACS and pathology examination of sections. All metastatic lymph nodes verified by pathology examination were also identified by FACS. Two additional lymph nodes with micro-metastases were diagnosed by FACS only.

**Conclusions:**

FACS analyses of pan-cytokeratin AE1/AE3 stained single cells from tumor draining lymph nodes can be used to detect micro-metastases in patients with penile cancer patients.

## Background

The concept of sentinel lymph nodes as the tumor draining lymph nodes was introduced by Cabanas in 1977 investigating patients with penile cancer [1]. It is widely accepted that the histological status of the regional lymph nodes in patients with solid tumors, including penile cancer, is an important predictor for patient survival [2–4]. Routine histopathology exam of penile cancer is performed by visual examination of hematoxylin and eosin (H&E)-stained slices under microscope by pathologists. However, this method is generally considered to be time-consuming and most importantly metastasis may be missed in this examination. For instance, one study in colon cancer found that 19.4% of lymph nodes, which contained micro-metastasis by immunohistochemistry (IHC), were not revealed by H&E examination [5]. Although IHC is not routinely used in penile cancer, it has been suggested that metastasis may also be missed in IHC, as evidenced by that examination of multiple IHC sections with automated computer-assisted image analysis detect additional metastasis [6], suggesting that examination of additional sections from every node increase the sensitivity [7]. However, both methods are extensive workloads for application in routine clinical practice. In addition, a 5 μm-thick section from a lymph node, which is normally examined in routine examinations, represents less than 0.1% of the node. Therefore, micro-metastasis may be missed in clinical pathology examination.

Our previous studies have demonstrated that flow cytometry can be used to detect micro-metastasis in lymph nodes from patients with colon, renal and head and neck cancer using cytokeratin antibodies [8–10]. In colon cancer the standard for IHC is to detect the presence of aberrant cytokeratin 20 staining in lymph nodes, while in renal cancer cytokeratin 18 is used to demonstrate metastasis. Moreover, it has been found that penile cancer cells are mainly keratinizing squamous cells [11], which express several cytokeratins including 1, 4, 5/6, 8, 13, 18, 19 and 20 [12]. Therefore, a Pan-cytokeratin reagent [13, 14], which is a cocktail of cytokeratin detecting antibodies, can be used to detect the presence of squamous cells in patients with penile cancer. Keratinizing squamous cells are found primarily in HPV negative penile cancer patients but to a less content in HPV positive patients [15]. Moreover, ectopic presence of epithelial cells in lymph nodes is considered as a result of metastasis [16]. These cells can be detected using EpCAM antibody which recognizes an adhesion molecule expressed on the cell surface of most epithelial cells [8]. In addition, the expression of E-cadherin, a type I cell adhesion molecule, has been associated with metastasis [17, 18].

In this paper, we evaluated the use of EpCAM, E-cadherin, and cytokeratins for detection of metastasis in tumor draining lymph nodes from penile cancer patients by flow cytometry.

## Methods

### Patients

Five patients with invasive penile cancer, 70–80 years old, from Södersjukhuset, Stockholm and Norrlands Universitetssjukhus, Umeå, Sweden, (2014–2015), were included (Table 1), with a preoperative staging; cT1-2 N0-1 M0. The study was approved by the regional ethical committee and written informed consent was obtained from all participants (EPN-Stockholm, dnr: 2013/835–32).

**Table 1:** Patient characteristics and lymph node detection

Patient	Age	Clinical tumor staging	pT- stages	Total no. of excised lymph nodes	Total no. of metastatic lymph nodes	Treatment after radical surgery	*Survival (months from the first surgery)
1	80	T2N0M0	pT2	7	0	Total amputation of penis 1.5 months later	Alive
2	77	T1N0M0	pT1	4	0	No	Alive
3	78	T2N1M0	pT2	8	1	No	Alive
4	78	T1N0M0	pT1a	4	2	Repeat Surgery 2 months later to remove bilateral lymph nodes inguinal and iliacal (yield 11/14 metastatic nodes)	4 months
5	70	T2N0M0	pT2	4	0	No	Alive

*Clinically determined during surgery. Alive, still alive at the latest follow-up in 6 August 2016

### Preparation of specimens

At surgery one piece of primary tumor was removed for the extraction of tumor cells as positive control. Lymph nodes (LNs) were identified and one half of LNs respectively underwent routine histopathology and immunological evaluation by flow cytometry. Venous blood was drawn and peripheral blood mononuclear cells (PBMC) were purified by ficoll-hypaque (Pharmacia, Amersham). Single cell suspensions from tissue specimens (if available to get) were obtained by gentle pressure using a loose-fit glass-homogenizer. Cells were washed twice and resuspended in AIM V® (Life technologies).

### Cell culture

The HeLa cell line CCL2 (ATCC) (HPV18 positive) was cultured in RPMI 1640 medium (Sigma) supplemented with 10% fetal bovine serum (FBS), 1% penicillin/streptomycin (Hyclone), and 1% L-glutamine (Hyclone). For flow cytometry detection, cells were detached with Trypsin-EDTA solution (Sigma).

### Identification of cancer cells in mixed cultures by flow cytometry

HeLa cells were added to PBMC, diluted in steps of three (3%, 1%, 0.33%, 0.11%, respectively), and kept in FACS-buffer (PBS containing 2% FCS and 0.05% NaN_3_). PBMCs alone were used as a negative control.

For surface staining, cells were washed and resuspended in FACS-buffer. Then cells were either directly labeled with an EpCAM antibody conjugated with PE (eBioscience) for 30 min at 4 °C, or incubated with an E-cadherin antibody (Dako) for 30 min at 4 °C, followed by incubation with goat-anti-mouse IgG conjugated with allphycocyanine (APC) (Jackson Immunoresearch) for 30 min in the dark.

For intracellular staining, cells were permeabilized with cytofix/cytoperm buffer (Becton Dickinson) for 30 min at 4 °C, washed and resuspended with 0.3% saponin (Sigma) in FACS-buffer. Direct labeling was performed by incubation of cells with anti-human pancytokeratin AE1/AE3 antibody conjugated with Alexa Fluor® 488 (eBiosience) for 30 min at 4 °C; indirect labeling was performed by incubation of cells with E-cadherin antibody or CK5/CK6 antibody (Dako), followed by incubation with goat-anti-mouse IgG conjugated APC for 30 min in the dark. An aliquot of the cells was stained with irrelevant conjugated antibodies as isotype controls.

Single-cell suspension from tumor draining lymph nodes was isolated by loose-fit glass homogenizer. Tumor cells were collected from tumor tissue using GentleMACS Dissociator (Miltenyi Biotec) in 10 ml RPMI 1640 medium (Sigma), containing 1% collagenase/Hyaluronidase solution (StemCell Technologies). Direct labeling with pancytokeratin AE1/AE3 antibody conjugated with Alexa Fluor® 488 was performed as above.

Cells were investigated using the LSR-FORTESSA (Becton Dickinson). Collected data were analyzed using FACS DIVA software (Becton Dickinson).

### Statistical evaluation

Comparisons between added and detected cells were evaluated by linear regression using the software GraphPad Prism. *P* value less than 0.05 was regarded as significant.

## Results

### Identification of tumor cells in mixed cultures using flow cytometry

**Fig. 1:**
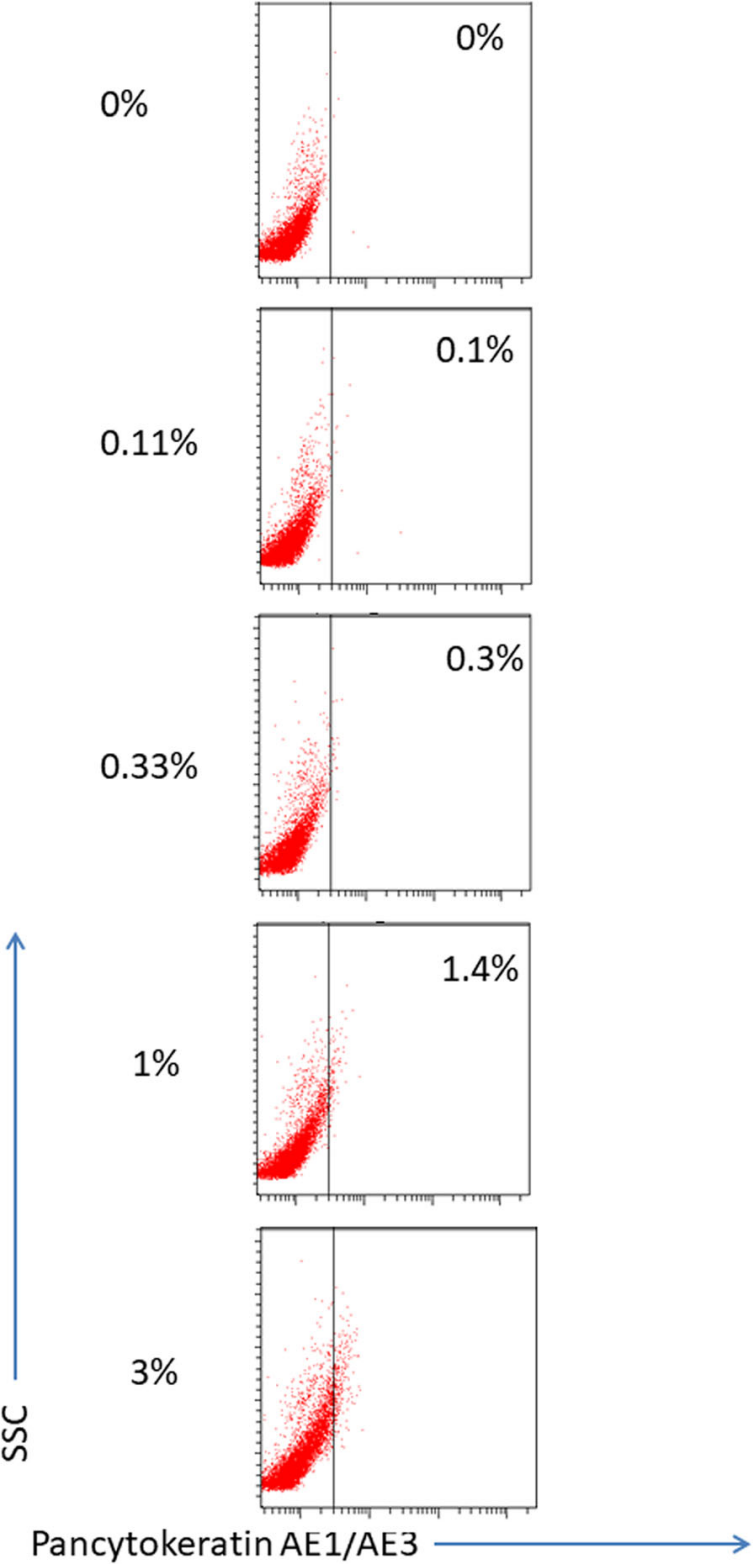
Detection of HeLa cells mixed with PBMCs. Hela cells were added to PBMCs and diluted in steps of three (3%, 1%, 0.33%, 0.11%, respectively), then stained with Pancytokeratin AE1/AE3 and detected by flow cytometry

To the best of our knowledge there are no penile cancer cell lines available. Since there is a resemblance between HPV positive penile cancer cells and cervical cancer cells [19] we decided to use HeLa cells for initial set up of the flow cytometry protocol. First, we tested HeLa cells for their cell surface expression of the epithelial marker EpCAM and E-cadherin, but no positive signal was found (data not shown). Next, we performed intracellular staining using E-cadherin and CK5/CK6 antibody. However again we failed to demonstrate any positive signal (data not shown). Therefore, we decided to use the pan-cytokeratin AE1/AE3 antibody mix which recognize subfamily A and B cytokeratins, and now we were able to detect a positive signal in HeLa cells compared to isotype control (data not shown). The HeLa cells in our cultures only expressed low amounts of cytokeratin allowing a stringent evaluation of the flow cytometry detection of tumor cells in a mixed leukocyte environment. Thus, in order to imitate the presence of metastatic cells in a lymph node we added decreasing number of HeLa cells into PBMCs from 3 to 0.11% in a serial dilution. When 3% HeLa cells were added, we detected 3.2% pan-cytokeratin AE1/AE3 positive cells (Fig. 1). HeLa cells were further titrated and when the lowest number of cells were added (0.11%) we detected 0.1% pan-cytokeratin AE1/AE3 positive cells in the mixed culture, demonstrating that the method can detect a small number of metastatic cells with precision and accuracy (Fig. 1).

### Stability of the method

**Fig. 2:**
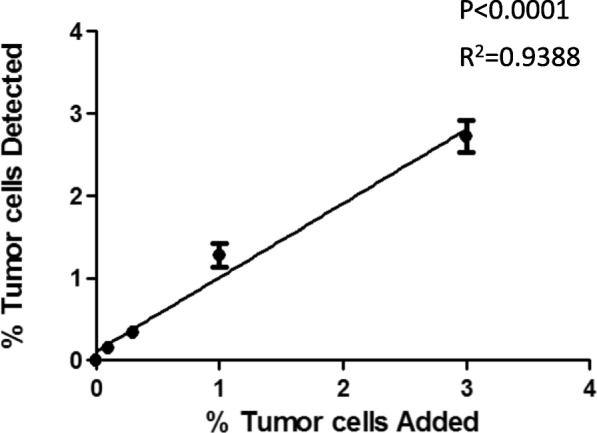
Sensitivity of flow cytometry detection assay. Y axis displayed the percentage of tumor cell added in the mixed cells. X axis demonstrate the percentage of tumor cells detected by flow cytometry. The linage regression analysis demonstrated a significant correlation between added and detected cells (*p* < 0.0001, r^2^ = 0.9388)

**Fig. 3:**
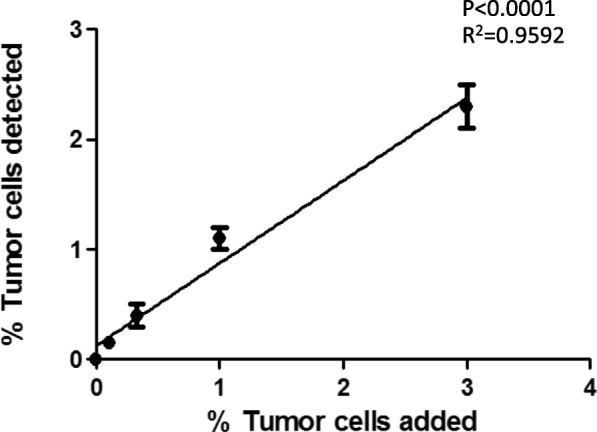
Stability of flow cytometry detection assay. To test for repeat measurement and stability over time, the same samples were run again after ~ 12 h. Y axis displayed the percentage of tumor cell added in the mixed cells. X axis demonstrate the percentage of tumor cells detected by flow cytometry. The comparison between added and detected cells demonstrated a significant correlation (*p* < 0.0001, r^2^ = 0.9592)

For evaluating the stability of the method we used PBMCs from 5 different donors, adding decreasing number of HeLa cells from 3 to 0.11%, and compared the number of added vs. detected pan-cytokeratin positive cells at five different occasions (Fig. 2). The linear regression analysis demonstrated a significant correlation between added and detected cells (*p* < 0.0001, r^2^ = 0.9388) (Fig. 2). The result indicates a linear and reliable detection of pan-cytokeratin positive cells from 0.11 to 3% of tumor cells in PBMCs. The detection of pan-cytokeratin positive cells demonstrated a good inter assay variability even when samples from different donors were used. To test for repeat measurement and stability over time, the same samples were run again after ~ 12 h. The comparison between added and detected cells demonstrated a significant correlation (*p* < 0.0001, r^2^ = 0.9592) (Fig. 3), indicating that the method is stable over time.

### Identification of Pan-cytokeratin positive cells from patients with penile cancer

**Fig. 4:**
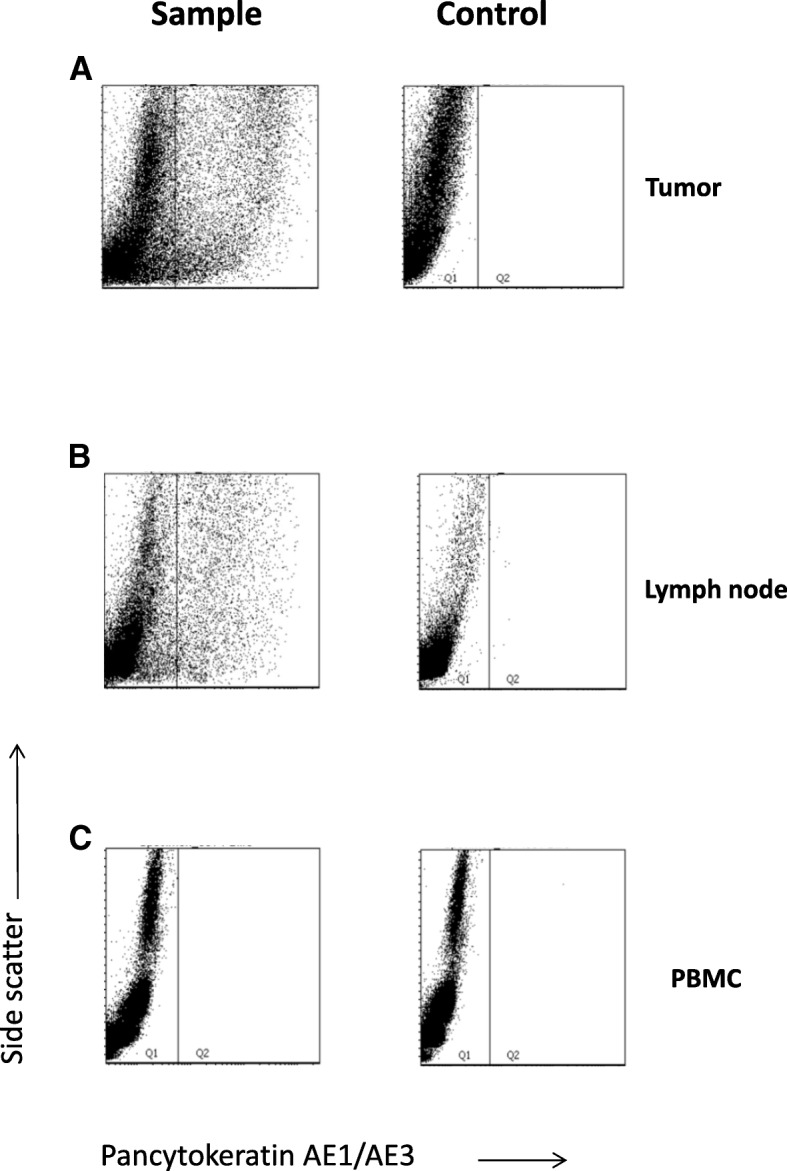
Detection of tumor cells using flow cytometry. Cell suspensions from the tumor (**a**), Lymph node (**b**) and peripheral blood mononuclear cells (PBMC) (**c**), from patient 9, were intracellularly stained with antibodies against Pancytokeratin AE1/AE3. Right panels were stained with isotype control

Next, we started to investigate the expression of pan-cytokeratin in single cells from penile tumors as a positive control (Fig. 4a). A large proportion of penile tumor derived cells were positive with a strong intracellular pan-cytokeratin staining when investigated by FACS (Fig. 4a). The pan-cytokeratin staining of penile cancer cells was in the order of 1–2 log stronger than seen in HeLa cells (Fig. 1).

A total of 10 lymph nodes were harvested from 5 patients with penile cancer (Table. 2). Lymph nodes were divided and sent for routine pathology and investigations with flow cytometry and intracellular pan-cytokeratin staining for a head to head comparison. We received one LN from patient 1 and we found 16.7% pan-cytokeratin-positive cells (Fig. 4b, left panel), demonstrating the presence of metastatic cells. When testing PBMCs from peripheral blood from the patients with penile cancer we were unable to detect any pan-cytokeratin positive cells, suggesting that no circulating metastatic cells were present, at least not above detection limit of 0.11% (Fig. 4c). Compared with the cells suspension from the tumor tissue, we found 47.6% pan cytokeratin positive cells (Fig. 4a). Two lymph nodes from patient 3 were investigated and we found 8.6% and 9.7% pan cytokeratin positive cells respectively (Table. 2) while we found one lymph node from patient 4 containing 2.1% pan cytokeratin positive cells (Table 2). Those lymph nodes were considered as metastatic lymph nodes.

**Table 2:** Investigation of PBMCs, Lymph nodes and cell suspensions extract from tumor tissues from patients with penile cancer

Patient No.	PBMC (%)	Lymph Node (%)	Cells suspension extract from tumor tissue (%)
1	0.4	0.2	ND
2	0.1	16.7	47.6
3	0.6	8.6	ND
3		9.7	
4	0.1	2.1	33.4
4		0.4	
4		1.1	
5	1.1	0.5	ND
5		0.1	
5		0.2	

In this study, we found Pan-cytokeratin-positive cells in a total of 4 LNs (4/10) (40%) from 3 of the 5 investigated penile cancer patients using flow cytometry (Table 3). In contrast routine pathology demonstrated metastatic cells in 3 LNs (3/17) from 2 out of 5 patients. Thus, the flow cytometry investigation using pan cytokeratin detection resulted in an upstaging of two patients, patients nos. 1 and 3. One LN from patient no.1 displayed Pan-cytokeratin-positive cells by FACS, whereas the pathology report was negative (pN0) in seven investigated LNs from the same patient. Thus, patient no. 1 was upgraded from pN0 to pN1. In addition, patient no. 3 was upgraded from pN1 to pN2 with FACS analysis. In patient no. 4 routine pathology identified 2 metastatic LN (2/4) whereas flow cytometry identified pan-cytokeratin positive cells only in 1 LN (1/3). Finally, in agreement with the pathology investigation patients 2 and 5 were staged as pN0 with both routine histopathology and flow cytometry (Table 3).

**Table 3:** Comparison of Pathology results and Pancytokeratin AE1/3 FACS results

Patient	Pathology results		pN staging (by standard pathology)	FACS results		pN staging (FACS)
	Total no. of ^a^LNs received	Total no. of metastatic LNs		Total no. of LNs tested	Total no. of metastatic LNs	
1	7	0	pN0	1	1	**pN1**
2	1	0	pN0	1	0	pN0
3	2	1	pN1	2	2	pN2
4^b^	4	2	pN2	3	1	pN1
5	3	0	pN0	3	0	pN0

^a^LNs stand for lymph nodes

^b^The second metastatic LN identified by pathology was never subjected to flow cytometry analysis

## Discussion

In this study, we investigated markers expressed by penile cancer cells using flow cytometry for identifying metastatic cells in the lymph nodes. We demonstrate that penile cancer cells could be detected in very small amounts. This detection method is sensitive and reliable. In our earlier studies, antibodies against CK20, EpCAM and CA19–9 were used to detect metastatic colon cancer cells and antibodies against CK18, CA9, and Cadherin 6 were used to detect renal cancer cells in lymph nodes [8, 9]. Here we add Pancytokeratin AE1/AE3 staining for detection of metastatic penile cancer cells to the list of solid tumors that may be evaluated for lymphatic dissemination by flow cytometry. Thus, tumors with specific markers, not present in lymph nodes, are likely candidates for flow cytometry based detection of metastatic cells.

The gold standard to evaluate lymph node metastasis is by pathology examination with or without out immunohistochemistry. However pathology examination is time consuming and costly since it involves technicians for preparation of sections and a skilled pathologist for ocular evaluation of the specimens. When using flow cytometry to detect metastasis in lymph nodes, the total time for preparation, staining of cells and evaluation is approximately 2-h were all the steps can be conducted by a technician. In addition, multiple samples can be prepared and analyzed at the same time. If compared with gold standard methodology, the flow cytometry assay is time saving and cost effective while maintaining sensitivity. There are many studies trying to improve the sensitivity in micro-metastasis detection. Some studies suggest detecting tumor antigen mRNA expression using the polymerase chain reaction (PCR) technique [20, 21]. These methods indeed improve the sensitivity of detection; however, they are time consuming and not easy to apply in clinical routine.

Since there are no penile cancer cell lines available, we used HeLa cells, a cervical cancer cell line which is HPV infected. Since studies on penile cancer found HPV negative tumors with keratinized cells expressing pancytokeratin AE1/AE3, there is a need to evaluate the method in HPV positive cases. Spiking Hela cells into PBMCs was used to estimate the accuracy of the metastasis detection in lymph nodes from patients with penile cancer. When we investigated the surface marker EpCAM and the cell adhesion marker E-cadherin, they both failed to detect tumor cells. From our cell spiking results, flow cytometry could detect as low as 0.11% of pancytokeratin AE1/AE3 stained tumor cells in the mixed leukocyte environment, demonstrating the method to be sensitive in detecting metastatic cells.

When applying our method to samples from penile cancer patients, FACS-results were in good agreement with routine pathology. However, two LNs were reclassified as metastatic due to presence of pan-cytokeratin positive cells identified by FACS. The results agree with our hypothesis: Since one section from a lymph node represents less than ~ 0.1% of the node [9] we assume that running single cell suspension through flow cytometry could obtain more information. In this small proof of concept study, we demonstrated that Pancytokeratin AE1/AE3 antibodies were useful for detecting metastatic penile cancer cells in lymph nodes. The technique to identify metastatic cells in lymph nodes from patients with penile cancer by staining for specific tumor markers and detection by flow cytometry needs to be systematically evaluated for sensitivity and specificity in a larger study where serial sectioning and pathology evaluation should be carried out head to head with flow cytometry based tumor specific marker identification.

## Conclusion

Intracellular pan-cytokeratin AE1/AE3 staining with flow cytometry can be used to detect micro-metastasis in tumor draining lymph nodes. Large scale head to head comparisons between FACS and routine pathology assessment are warranted.

## Abbreviations

FACS: Fluorescence-activated cell sorting
H&E: Hematoxylin and eosin
IHC: Immunohistochemistry
LNs: Lymph nodes
PBMC: Peripheral blood mononuclear cells
PCR: Polymerase chain reaction
